# Effects of spawning Pacific salmon on terrestrial invertebrates: Insects near spawning habitat are isotopically enriched with nitrogen‐15 but display no differences in body size

**DOI:** 10.1002/ece3.8017

**Published:** 2021-08-24

**Authors:** Nicola F. Rammell, Allison M. Dennert, Christopher M. Ernst, John D. Reynolds

**Affiliations:** ^1^ Earth to Ocean Research Group Department of Biological Sciences Simon Fraser University Burnaby BC Canada; ^2^ Hakai Institute Heriot Bay BC Canada

**Keywords:** body condition, body size, Carabidae, Curculionidae, insects, isotope, marine‐derived nutrients, nitrogen, Pacific salmon

## Abstract

When Pacific salmon (*Oncorhynchus* spp.) spawn and die, they deliver marine‐derived nutrient subsidies to freshwater and riparian ecosystems. These subsidies can alter the behavior, productivity, and abundance of recipient species and their habitats. Isotopes, such as nitrogen‐15 (^15^N), are often used to trace the destination of marine‐derived nutrients in riparian habitats. However, few studies have tested for correlations between stable isotopes and physiological responses of riparian organisms. We examined whether increases in *δ* ^15^N in terrestrial insect bodies adjacent to salmon spawning habitat translate to changes in percent nitrogen content and body size. This involved comparisons between distance from a salmon‐bearing river, marine‐derived nutrients in soils and insects, soil moisture content, and body size and nitrogen content in two common beetle families (Coleoptera: Curculionidae, Carabidae). As predicted, δ^15^N in riparian soils attenuated with distance from the river but was unaffected by soil moisture. This gradient was mirrored by δ^15^N in the herbivorous curculionid beetles, whereas carabid beetles, which feed at a higher trophic level and are more mobile, did not show discernable patterns in their δ^15^N content. Additionally, neither distance from the river nor body δ^15^N content was related to beetle body size. We also found that nitrogen‐15 was not correlated with total percent nitrogen in insect bodies, meaning that the presence of spawning salmon did not increase the percent nitrogen content of these insects. We conclude that while salmon‐derived nutrients had entered terrestrial food webs, the presence of δ^15^N alone did not indicate meaningful physiological changes in these insects in terms of percent nitrogen nor body size. While stable isotopes may be useful tracers of marine‐derived nutrients, they cannot necessarily be used as a proxy for physiologically important response variables.

## Graphical abstract

Pacific salmon are ecologically important to plant and animal species in coastal ecosystems along western North America, northeastern Asia and beyond. This paper examines the relationship between spawning salmon and riparian beetle species to determine whether salmon‐derived nutrients can influence body size and nutrient composition in insects. We found that while salmon‐derived nutrients had entered terrestrial food webs, the presence of these nutrients alone did not indicate meaningful physiological changes in these insects in terms of percent nitrogen nor body size.

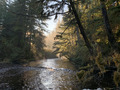

## 1 INTRODUCTION

The exchange of nutrients between marine and terrestrial ecosystems can have a wide variety of biological consequences (Fariña et al., 2003; Nakano & Murakami, 2001; Walsh et al., 2020). Nutrient exchange across ecosystem boundaries can occur through the movement of nutrients by passive forces such as wind and water, or by active biotic forces such as animal migration (Fariña et al., 2003; Polis et al., 1997). When nutrients and materials move from more productive to less productive habitats, these nutrients provide nutrient subsidies. Subsidies from animal migration are of particular interest in coastal ecosystems with anadromous fishes, where nitrogen and phosphorus subsidies can trigger bottom‐up effects on primary productivity and invertebrate abundance and biomass (Helfield & Naiman, 2001; Hocking et al., 2013; Wipfli et al., 1999).

In coastal regions of the North Pacific, spawning migrations of Pacific salmon (Haíɫzaqvḷa [Heiltsuk language]: miá) (Latin: *Oncorhynchus* spp.) deliver annual pulses of subsidies from marine to terrestrial ecosystems in large quantities each year. After growing at sea, salmon migrate to their natal freshwater streams to spawn. Though many populations spawn near the coasts, some travel over 1,000 km upstream (Groot & Margolis, 1991; Quinn, 2018). Most species of Pacific salmon die after spawning, and their carcasses can be moved into riparian habitats by terrestrial scavengers and predators, including bears (náṇ or ƛ̓á) (*Ursus* spp.) and wolves (k̓vsḷs) (*Canis lupis*) (Harding et al., 2019). Additionally, whole carcasses and salmon‐derived nutrients may be deposited into terrestrial habitat via flooding and subsurface flow (Ben‐David et al., 1998; Cederholm et al., 1989; O'Keefe & Edwards, 2002). Decomposing carcasses can then contribute to the nutrient budgets of riparian areas, which are frequently limited in nitrogen (Darimont et al., 2010; Hocking & Reimchen, 2002), with a wide variety of effects on freshwater and terrestrial communities (Janetski et al., 2009; Walsh et al., 2020).

Marine‐derived nutrients are often traced to riparian organisms using stable isotopes, especially of nitrogen (^15^N), in a variety of taxa and terrestrial food chains (Bilby et al., 1996, Ben‐David et al., 1998, Reimchen et al., 2002; reviewed by Walsh et al., 2020). For example, previous work has shown that nitrogen‐15 in riparian soils, invertebrates, and vertebrate species is positively correlated with the presence and abundance of salmon in adjacent freshwater rivers. This has been shown both experimentally in plants (Hocking & Reynolds, 2012) and through correlations involving mosses, liverworts, and a variety of riparian shrub and forb species (Hocking & Reynolds, 2011; Wilkinson et al., 2005). Studies of terrestrial litter invertebrates and birds indicate isotopic enrichment (Christie et al., 2008; Hocking & Reimchen, 2002; Reimchen et al., 2002), as well as changes in density and diversity in both taxa (Christie & Reimchen, 2005; Field & Reynolds, 2011; Hocking et al., 2009; Reynolds & Wagner, 2019).

Despite extensive study of salmon‐derived nitrogen‐15 enrichment in riparian systems, the effects of this enrichment on the survival and reproduction of riparian organisms are often poorly understood. Previous work has focused on community‐level impacts of nitrogen enrichment and has captured changes in abundance and diversity of riparian species alongside salmon‐bearing streams (Hocking et al., 2013; Hocking & Reynolds, 2011; Wagner & Reynolds, 2019) and on estimations of the proportion of salmon‐derived nitrogen in the organisms’ percent nitrogen content (e.g., Bilby et al., 2003; Helfield & Naiman, 2001). While these estimates approximate the extent to which nutrients from salmon are incorporated into organismal tissues, they do not inform us of resulting biological consequences.

In terrestrial insects, body size is strongly related to survival and reproduction and is commonly used as a proxy for body condition. For example, in female beetles, body size is highly correlated with fecundity (Honek, 1993; Juliano, 1985; Kajita & Evans, 2010; Stillwell & Fox, 2009). Large male beetles hold a competitive advantage against smaller rivals and are more likely to mate with large females (Fox et al., 2007; Juliano, 1985). Adult insect body size and thus reproductive potential are determined by larval growth, which reflects environmental conditions such as habitat quality (Barone & Frank, 2003; Bommarco, 1998) and food availability during early development (Chown & Gaston, 2010). Additionally, fecundity and larval development are positively correlated with host plant nitrogen and body nitrogen in a variety of insect species (Awmack & Leather, 2002; Ohmart et al., 1985; Riedell et al., 2011). Thus, body size is a simple and robust metric of body condition. To our knowledge, only one study has attempted to link salmon‐derived nutrients to body condition of terrestrial invertebrates and they found that condition was not correlated with stable isotope patterns in wood ants (*Formica* spp.) or wolf spiders (*Pardosa* spp.) (Vizza et al., 2017).

**FIGURE 1:**
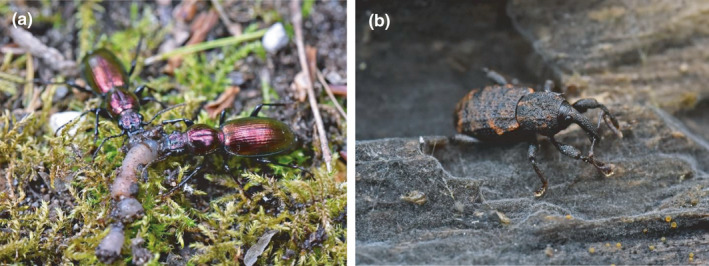
(a) *Zacotus mathewsii* and (b) *Steremnius carinatus*, representing ground beetles (Coleoptera: Carabidae) and weevils (Coleoptera: Curculionidae), respectively (Photo credit: Sung Jo)

We sampled terrestrial insects on the banks of a salmon‐bearing river on the central coast of British Columbia, Canada. We tested for correlations between δ^15^N and body size for two beetle (páɫsṃ́) families: weevils (Coleoptera: Curculionidae) and ground beetles (Coleoptera: Carabidae) (Figure 1). These taxa represent herbivores and carnivores, respectively, thereby allowing a test for links between marine‐derived nutrients and a metric of body condition at two trophic levels. We traced nitrogen‐15 using stable isotope ratios, and we used insect body size as a physical response metric while accounting for potential confounds such as soil moisture. We tested for evidence of increased body size (using elytron length) in an ecosystem that receives marine subsidies by asking the following questions (Table 1): First, does soil δ^15^N increase with proximity to a salmon‐bearing river? Second, does insect body δ^15^N increase with proximity to the river, and does this result in an increase in percent nitrogen content? Third, are insects with elevated body δ^15^N larger? And lastly, are larger insects found closer to the river? We hypothesized that patterns of marine‐derived nutrients would follow a gradient in both the soil and the terrestrial insects with distance from the salmon‐bearing river, and tested whether near‐river nitrogen enrichment translates to increased body size for weevils and ground beetles.

## 2 MATERIALS AND METHODS

### 2.1 Study system

**FIGURE 2:**
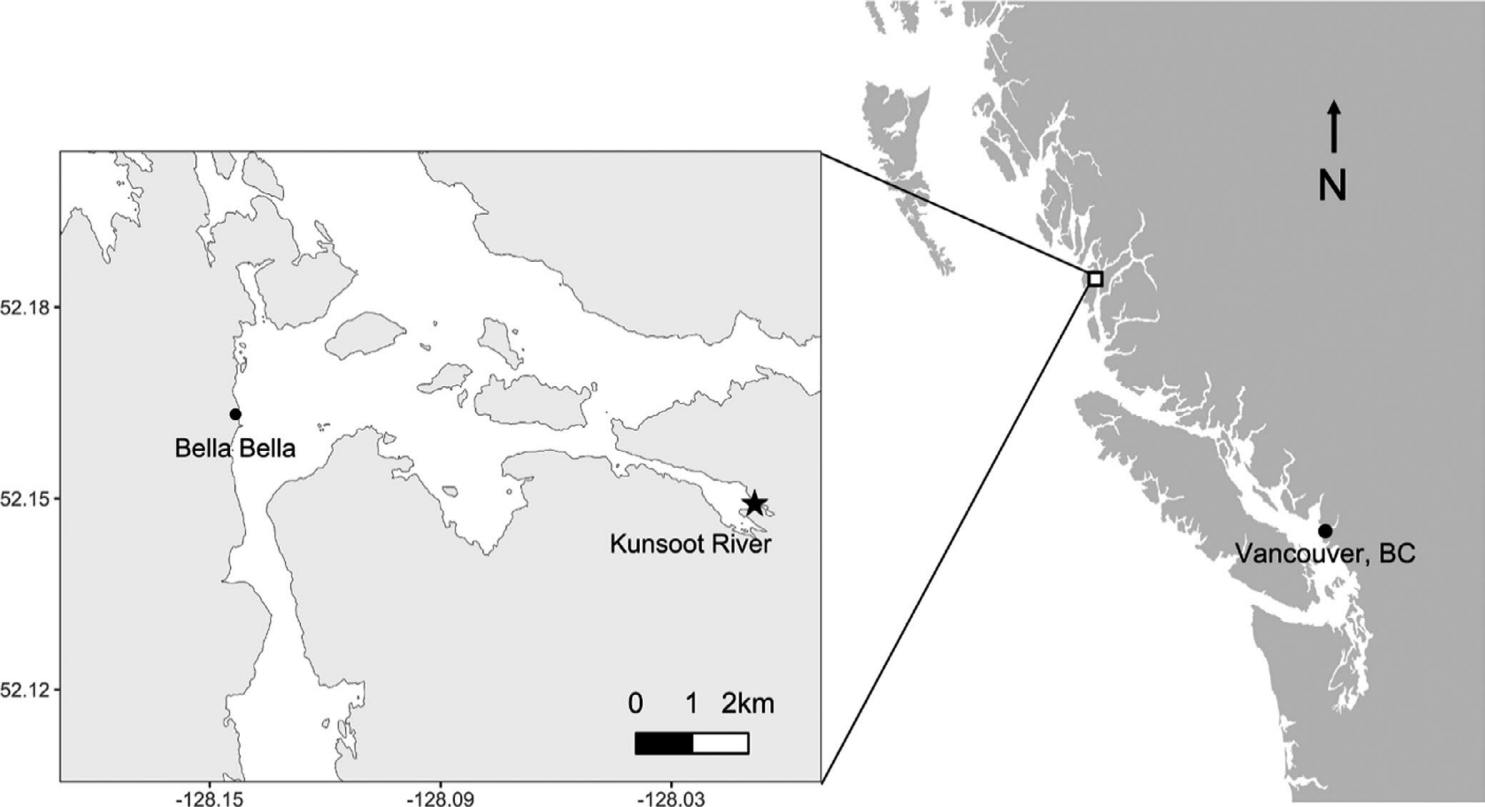
Study area along the Q̓íɫcutkv (Kunsoot) River (N52°8′50.8″, W128°0′10.9″) near Bella Bella on the central coast of British Columbia, Canada

**FIGURE 3:**
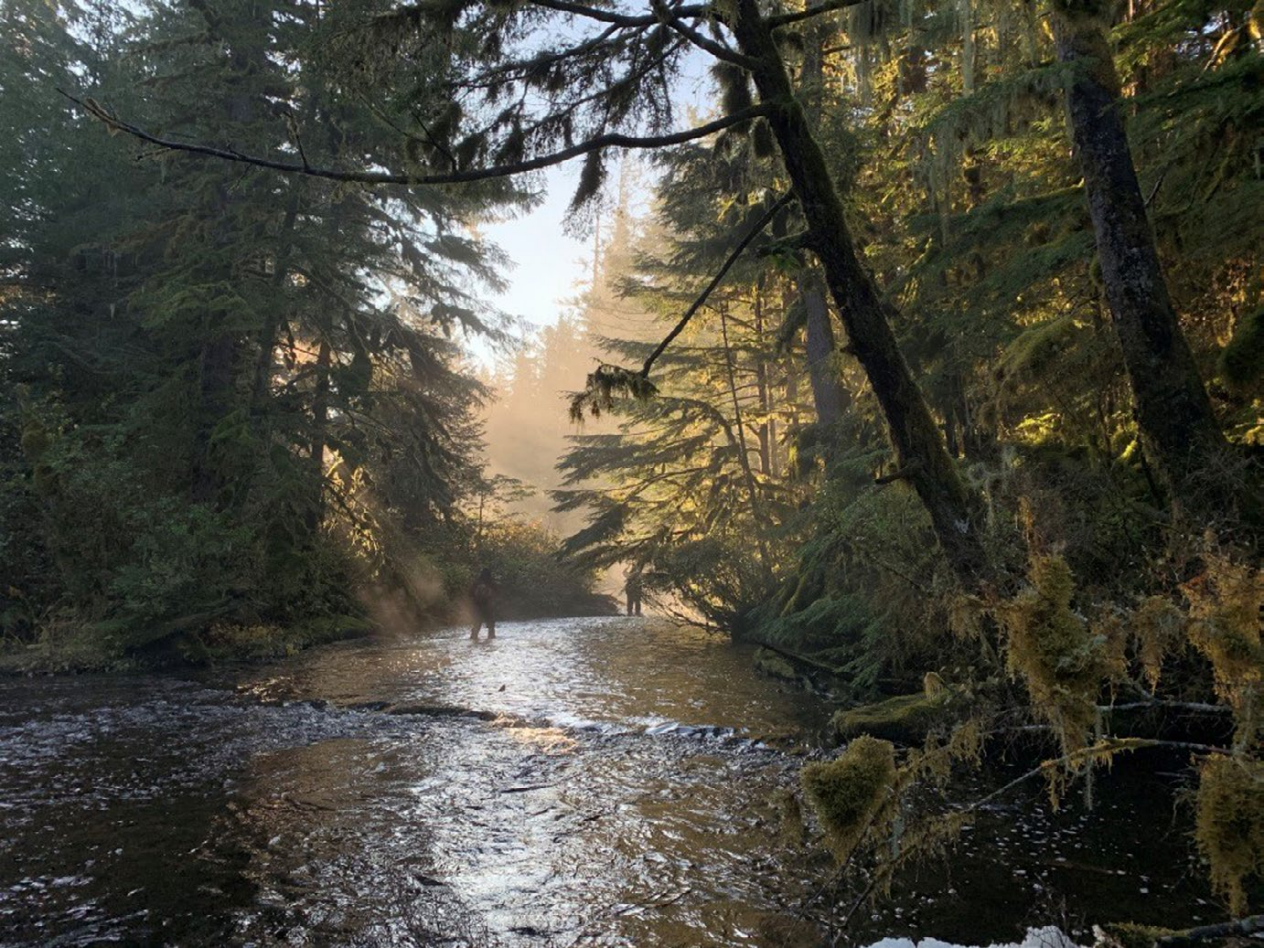
The study area along the first 180 m of the Q̓íɫcutkv (Kunsoot) River. Pitfall traps were set in the riparian area on the left bank (Photo credit: Emily Yungwirth)

We conducted this study in the territory of the Haíɫzaqv (Heiltsuk) First Nation along a bank of the Q̓íɫcutkv (Kunsoot) River (N52°8′50.8″, W128°0′10.9″), on the central coast of British Columbia, near Wágḷísḷa (Bella Bella) (Figures 2 and 3). This river supports well‐documented spawnings of pink (k̓áp̓i) (*Oncorhynchus gorbuscha*) and chum (ĝváx̌ṇís) (*Oncorhynchus keta*) salmon, until approximately 900 m upriver where a small waterfall acts as a barrier to fish. Occasional individual sockeye (hísṇ) (*Oncorhynchus nerka*) and coho salmon (zu̓ṇ́) (*Oncorhynchus kisutch*) frequent the river as well. The fish spawn from late August to early November. The average three‐year (2015–2017) peak spawning numbers of chum and pink salmon are approximately 4,200 and 6,200 individuals, respectively (Reynolds Lab, unpublished data). We worked in the forest on the north side of the first 180 m of the river, a relatively straight channel with a bankfull width of 15.1 m (Figure 3). This region is in the Coastal Western Hemlock Biogeoclimatic Zone (Pojar et al., 1987) and is characterized by nutrient‐poor soils, high annual precipitation (>3,000 mm annually), and a mean annual temperature of about 8°C.

We examined curculionid weevils and carabid ground beetles, which represent two different trophic levels in riparian ecosystems. The weevils in our study area (*Steremnius* spp.) establish their broods on young conifer seedlings from spring to late summer, and larvae feed within the phloem until winter. Newly eclosed adult weevils emerge in mid‐late summer and feed on a variety of riparian plants such as huckleberry (ĝvádṃ) (*Vaccinium* sp.), salal (nk̓as) (*Gaultheria shallon*), fireweed (*Epilobium angustifolium*), and various conifers, then cease feeding in the fall at the onset of frosty weather (Condrashoff, 1968). Ground beetles are active generalist predators that feed on herbivorous and detritivorous insects during the summer months, as well as on fly eggs and larvae deposited on salmon carcasses in the fall (Hocking et al., 2009). They also known to feed on slugs, snails, isopods, worms, and springtails (Hocking & Reimchen, 2002), but are likely not a direct consumer of salmon carcasses (Reimchen et al., 2002). They survive at least one winter to breed in the spring (Niwa & Peck, 2002).

### 2.2 Insect collection and soil sampling

We used round, white plastic pitfall traps (11.5 cm diameter, 8 cm tall) to collect weevils and ground beetles. We delineated 10 transects running perpendicular to the river, spaced 20 m apart. Along each of the 10 transects, collection stations were marked at distances 0, 25, and 50 m away from the river, for a total of 30 collection stations forming a grid. The 0 m collection stations were located as close to the river as possible, while avoiding flooding at high bankfull (≤5 m from the channel). At each of the 30 collection stations, we haphazardly set three pitfall traps within 1 m of each other, for a total of 90 pitfall traps deployed. Traps were buried so their rims were flush with the ground, and we added a drop of surfactant and approximately 125 ml of propylene glycol to preserve the insects. At each station, we also collected a soil sample from the location of each trap (10 cm below ground), which was transported to the laboratory and frozen for later soil moisture and isotope analysis.

All beetles were collected in July 2018, with traps serviced every six days, four times (5–6 July, 11–12 July, 17–18 July, and 23–24 July). The summer is the height of the growing season for riparian plants and the most active feeding period of the insects we studied. After the fall spawning events, salmon‐derived nutrients are stored in the soil and soil microbiota over winter and taken up by plants to use in foliar tissues in the following spring and summer. Larval weevil feeding would begin in the spring, and adult feeding on plant tissues is highest in spring and summer. Similarly, ground beetles feed on live invertebrate prey in the warmest months and their diets and nitrogen contents would mostly be affected by the food sources of their predominantly herbivorous and detritivorous prey. Because we retrieved fewer insects than anticipated after the first 2 collections, and we were not measuring abundances, we increased the number of traps at each collection station from three to five for the second half of the sampling period, for a total of 150 traps deployed. We sorted pitfall trap samples in the field, using forceps to retrieve all weevils and ground beetles, placing specimens immediately into vials of 95% ethanol, which were transported to the laboratory for pinning and identification. In some cases, preservatives can affect isotopic analyses, but have been shown to affect δ^15^N values with less frequency and magnitude than other values, such as δ^13^C (Kaehler & Pakhomov 2001; Sarakinos et al., 2002).

In the laboratory, weevils and ground beetles were cleaned in 95% ethanol using forceps to remove dirt and debris, pinned, and dried at room temperature. We identified all weevils and ground beetles to species using a reference collection and traditional morphological features (Lindroth, n.d., Hatch, 1971). All ground beetles were sexed using foreleg tarsus features, which are enlarged and hairy on male specimens (Holliday, 1977), except those destroyed in stable isotope analysis due to logistical and temporal constraints. We could not determine the sexes of weevils due to the lack of sexual dimorphism in the species we collected. Thus, sex is only included in statistical analyses of carabid specimens that were not sent for isotope analyses. We measured elytron length of all specimens using digital calipers (to the nearest 0.01 mm), as that is a commonly used metric to measure body size in beetles (Knapp & Knappová, 2013). One observer independently measured all specimens three times to test the repeatability of the measurements (see Section 2.4).

### 2.3 Stable isotope analysis

We randomly selected a subset of focal insects consisting of one ground beetle and three weevils from each distance along each transect (i.e., each station) for stable isotope analysis. We chose the most common ground beetle species, *Zacotus matthewsii* LeConte, 1869, as a focal species because at least one individual was collected from each station (Figure 1). We chose the most common weevil species, *Steremnius carinatus* Boheman, 1842, which was collected from all but two stations, as the weevil focal species (Figure 1). Because of their small size (elytron length ~4 mm), three *S*. *carinatus* specimens from each station were pooled into one sample to ensure sufficient material for analysis. These whole insect specimens, already air‐dried on pins, were dried at 60°C for 24 hr before we ground them into a fine, homogeneous powder using a Wig‐L‐Bug grinder and packaged 0.5–1.0 mg into aluminum capsules for analysis.

All soil and insect samples were analyzed at the Pacific Forestry Centre Analytical Chemistry Laboratory, Canadian Forest Service, in Victoria, British Columbia. Fresh soil samples were kept frozen at −10°C until they could be analyzed. The three replicate soil samples from each of the 30 stations were first combined to form one sample from each station. Soil samples were then dried at 70°C, ground, and homogenized for stable isotope analysis. Moisture content of wet soils (moisture over dry weight (g/g)) was also recorded to account for potential confounds in isotopic ratios, as soil moisture can affect fractionation of nitrogen isotopes (Naiman et al., 2002). Relative abundance of nitrogen and carbon isotopes was measured using the Thermo Scientific Delta V Advantage Isotope Ratio Mass Spectrometer with the Flash 2000 Organic Elemental Analyzer. Percent nitrogen (calculated as total nitrogen over dry sample mass) was also derived during this analysis. We examined isotopic ratios of nitrogen, calculated using the formula δ^15^N(‰) = (*R*_sample_/*R*_standard_ – 1) × 1,000, where *R* is the ratio of the heavy isotope (^15^N) to the light isotope (^14^N) (Fry, 2006). Data were normalized using the measured and theoretical values of two reference materials. Isotopic values are abbreviated using delta notation, δ, and expressed as ratios in parts per thousand (‰).

We also calculated an approximate percentage of putative salmon‐derived nitrogen in the bodies of both weevils and ground beetles collected 0 m away from the stream. We used equations adapted from Vizza et al. (2017) and Hocking and Reimchen (2002), as well as data from Satterfield and Finney (2002) on the δ^15^N of chum salmon carcass tissue (11.01‰). We elected to use chum carcass tissue for this calculation as chum make up the majority of spawner abundance by biomass, and this number was statistically indistinguishable from pink salmon δ^15^N (10.79‰). This approximation necessarily assumes no influence of carcasses 50 m away from the stream as it is used as a baseline, and the consequences of this assumption are discussed.

### 2.4 Statistical analyses

All analyses were conducted in R, version 3.5.1 (R Core Team, 2018). We used the lm function in base R and the lmer function in the lme4 package (Bates et al., 2015) to generate linear models and linear mixed‐effects models. To test the repeatability of the three replicate elytron length measurements for each individual insect, we fit length measurements as a function of individual insect identification, which was included as a random effect. We verified residuals for normality and homogeneity of variance. We used the VarCorr function in the lme4 package to extract the estimated variance components and calculate a repeatability factor, *R* = *σ* ^2^_among_/(*σ* ^2^_among_ + *σ* ^2^_within_), to quantify the repeatability of triplicate measurements. After verifying high repeatability (*R* = 0.999), indicating very low within‐insect variation relative to between‐insect variation, we selected the median measurement of elytron length for each insect for subsequent analyses.

We created models to explore each of our questions, with models run separately for weevils and ground beetles (Table 1). All variables were included as fixed effects unless otherwise stated, and we allowed the effects to interact in some explicit cases. To test for a soil δ^15^N gradient, we fit soil δ^15^N as a function of distance from river and soil moisture, allowing distance and moisture to interact. Distance and soil moisture were scaled and centered to standardize continuous variables on different scales. We tested for a correlation between distance and moisture using Pearson's correlation coefficient, *r*. Using the subset of insects analyzed for stable isotopes, we fit models to describe patterns of insect body δ^15^N and as a function of distance from the river. Using this same subset of insects, we fit models to describe percent nitrogen and insect body size as a function of insect body δ^15^N. For the weevils, elytron lengths were averaged across the three insects pooled for isotope analysis. For these stable isotope models, transect (or distance upstream) was not included since data collection was constrained to a distance of 180 m upstream and inclusion resulted in a singular model fit due to constraints on the number of insects of each species captured at each collection station. Using the full dataset, we fit a model to describe insect body size as a function of distance from river, species, sex (in the case of ground beetles), sampling week, and transect. Ground beetles destroyed in stable isotope analysis were not included in this model as their sexes were unknown. Transect was included as a random effect, whereas sampling week, with only four levels, was included as a fixed effect. Distance and sampling week were standardized. Distance and species were allowed to interact because of an a priori hypothesis that distance may affect insect body size differently by species.

**TABLE 1:** Research questions and corresponding statistical analyses with sample sizes

Research question	Model	Sample size
1	Soil δ^15^N ~ distance from river * soil moisture	*n* = 30
2	Weevil body δ^15^N ~ distance from river	*n* = 28
2	Ground beetle body δ^15^N ~ distance from river	*n* = 30
2	Weevil body %N ~ body δ^15^N	*n* = 28
2	Ground beetle body %N ~ body δ^15^N	*n* = 30
3	Weevil body size ~body δ^15^N	*n* = 28
3	Ground beetle body size ~body δ^15^N	*n* = 30
4	Weevil body size ~distance from river * species +sampling week + (1| transect)	*n* = 1,010
4	Ground beetle body size ~distance from river * species +sex + sampling week + (1| transect)	*n* = 290

Note

Reported are the models corresponding to the following research questions: (1) Does soil δ^15^N increase with proximity to a salmon‐bearing river? (2) Does insect body δ^15^N increase with proximity to the river, and does this result in an increase in *%N* content? (3) Are insects with elevated body δ^15^N larger? (4) Are larger insects found closer to the river?

For all models, we verified residuals for normality and homogeneity of variance and checked for outliers, overdispersion, and model fit using the DHARMa package (Hartig, 2019). For linear models, we used the summary function in base R to report the multiple *R* ^2^, *F*‐statistic, *df*, and *p*‐value. For the full linear mixed model, we extracted coefficient estimates, standard error, and *t*‐values using the summary function in base R (Table 2).

**TABLE 2:** Parameter estimates for fixed effects where *n* = number of observations, Parameter = explanatory variable, Value = coefficient estimate, and *SE* = standard error. Transect was included as a random effect

Response variable	*n*	Parameter	Value	*SE*	*t*‐Value
(a) Weevil body size	1,010	**Distance**	0.018	0.045	0.402
		**Species**			
		*Steremnius carinatus*	−2.242	0.046	−48.6
		**Distance × species**			
		*Steremnius carinatus*	−0.003	0.048	−0.062
		**Sampling week**	0.002	0.017	0.148
(b) Carabid body size	290	**Distance**	−0.040	0.075	−0.530
		**Species**			
		*Pterostichus crenicollis*	2.870	0.144	19.9
		*Scaphinotus angusticollis*	6.043	0.116	52.3
		*Zacotus matthewsii*	2.491	0.088	28.4
		**Distance × species**			
		*Pterostichus crenicollis*	0.044	0.134	0.331
		*Scaphinotus angusticollis*	−0.012	0.112	−0.107
		*Zacotus matthewsii*	0.028	0.084	0.339
		**Sex**	−0.205	0.063	−3.28
		**Sampling week**	−0.001	0.030	−0.031

**TABLE 3:** Number of weevil (Coleoptera: Curculionidae) and ground beetle (Coleoptera: Carabidae) specimens collected from pitfall traps at three distances away from the river over the sampling period

	Distance from river (m)			Total
	0	25	50	
**Curculionidae**				
*Steremnius carinatus*	277	263	300	840
*Steremnius tuberosus*	69	66	35	170
	346	329	335	1,010
**Carabidae**				
*Pterostichus amethystinus*	7	17	39	63
*Pterostichus crenicollis*	21	5	3	29
*Scaphinotus angusticollis*	9	10	15	34
*Zacotus matthewsii*	75	58	63	196
	112	90	120	322

## 3 RESULTS

We collected, measured, and identified 1,338 weevils and ground beetles (Table 3). There were two abundant weevil species, *S*. *carinatus* and *S*. *tuberosus* Gyllenhal, 1835, and four abundant ground beetle species, which in order of decreasing abundance, were *Z*. *matthewsii, Pterostichus amethystinus* Mannerheim, 1843*, Scaphinotus angusticollis* (Mannerheim, 1823), and *P*. *crenicollis* LeConte, 1873. We dropped one weevil species (*Nemocestes incomptus* [Horn, 1876] [*n* = 1]) and two ground beetle species (*Leistus ferruginosus* Mannerheim, 1843 [*n* = 2], *Cychrus tuberculatus* Harris, 1839 [*n* = 3]) from our analyses due to insufficient sample sizes. Sample sizes were similar at 0, 25, and 50 m from the river (Table 3).

**FIGURE 4:**
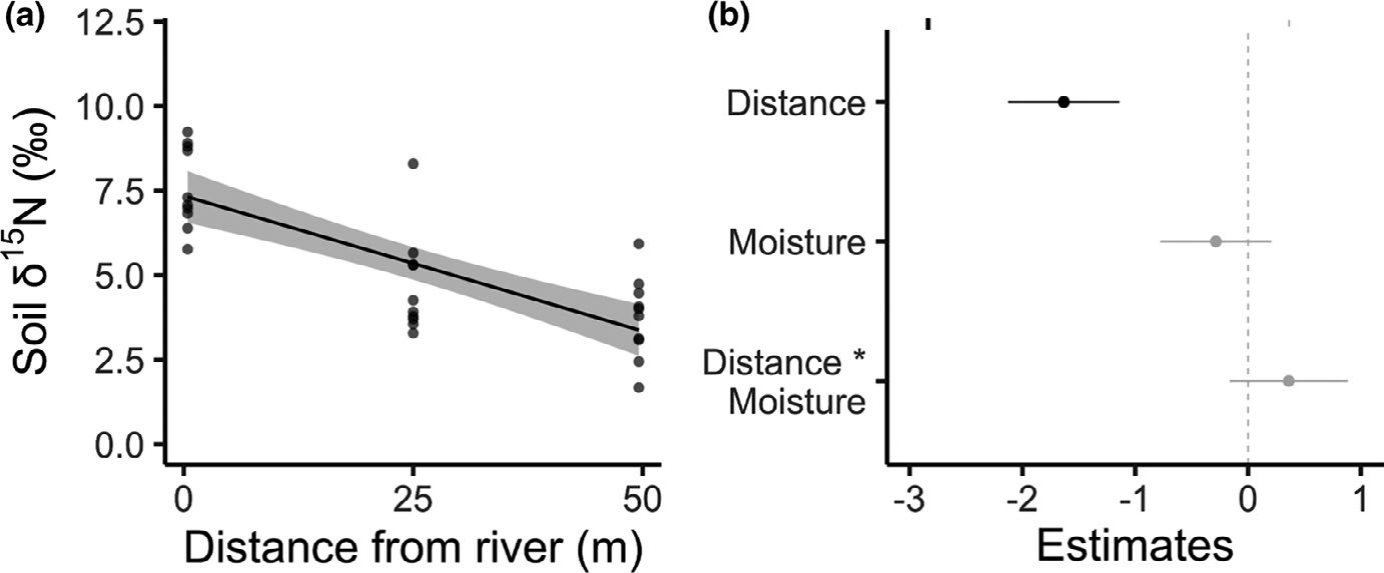
(a) Soil δ^15^N in relation to distance from the river. Points indicate raw data, and the line represents model predictions with 95% confidence intervals while accounting for soil moisture and its interaction with distance from the river. (b) Coefficient plot for the soil δ^15^N model, which shows each parameter estimate with 95% confidence intervals. Coefficients with confidence intervals that do not cross zero are shown in black

As expected, soil δ^15^N levels were highest near the river and decreased with distance (multiple *R* ^2^ = 0.63, *F*‐statistic = 14.6, *df* = 26, *p*_distance_ < .0001; Figure 4). Soil moisture and its interaction with distance had no significant relationship to soil δ^15^N (*p*_moisture_ = .266, *p*_moisture*distance_ = .189). Soil moisture ranged from 1.52 to 7.41 g/g (moisture over soil dry weight), consistent with high rainfall and soil organic matter content in the study region. Soil moisture showed no discernable spatial patterns. Variance in soil δ^15^N was more strongly explained by distance from the river. Distance from the river and soil moisture were not correlated (*r* = −0.006).

**FIGURE 5:**
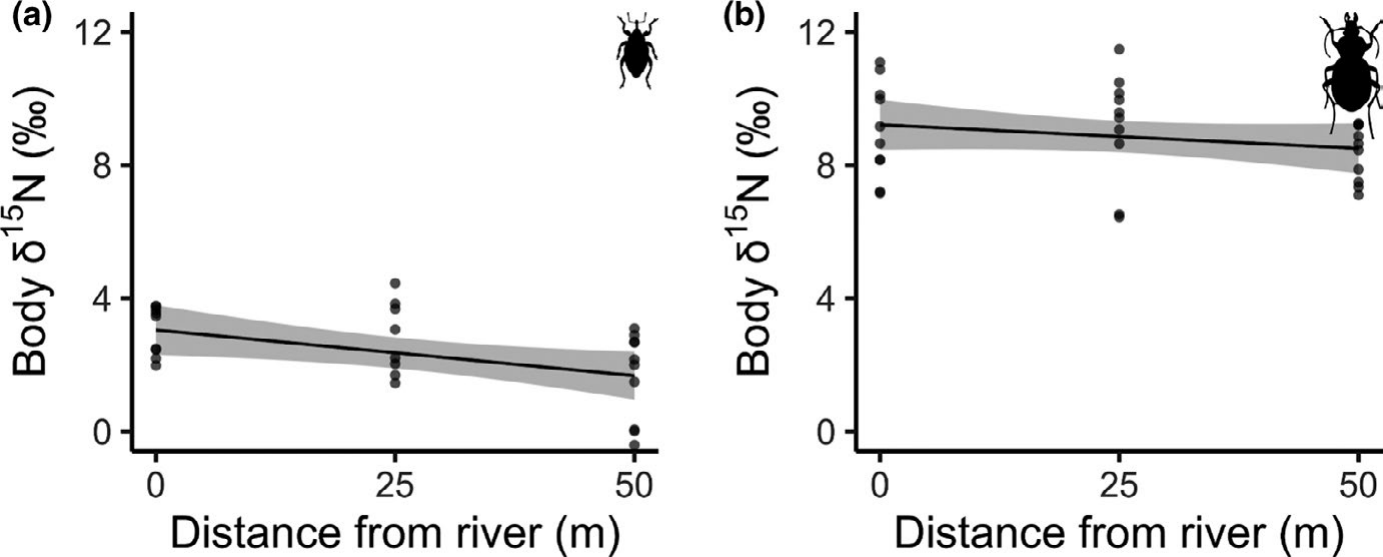
(a) Weevil (*Steremnius carinatus*) body δ^15^N in relation to distance from the river. (b) Ground beetle (*Zacotus matthewsii*) body δ^15^N in relation to distance from the river. Points indicate raw data, and the line represents model predictions with 95% confidence intervals

Weevil body δ^15^N also decreased with distance from the river (multiple *R* ^2^ = 0.178, *F*‐statistic = 5.6, *df* = 26, *p* = .025; Figure 5a), but there was no significant relationship for ground beetle δ^15^N (multiple *R* ^2^ = 0.049, *F*‐statistic = 1.4, *df* = 28, *p* = .242; Figure 5b). Ground beetles were consistently higher in body δ^15^N than weevils, as expected, from their higher trophic position. We also found that body ^15^N enrichment did not result in elevated body percent nitrogen for either weevil (multiple *R* ^2^ = 0.093, *F*‐statistic = 2.7, *df* = 26, *p* = .115) or ground beetle (multiple *R* ^2^ = 0.053, *F*‐statistic = 1.6, *df* = 28, *p* = .222).

**FIGURE 6:**
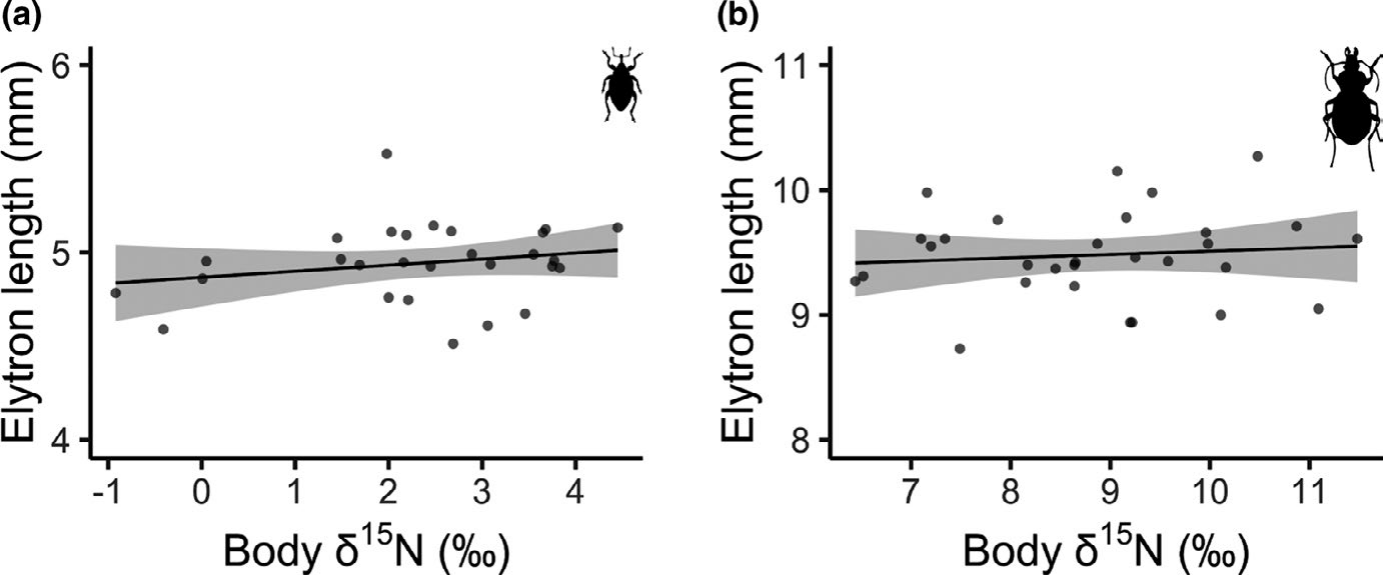
(a) Weevil (*Steremnius carinatus*) elytron length in relation to body δ^15^N. (b) Ground beetle (*Zacotus matthewsii*) elytron length in relation to body δ^15^N. Points indicate raw data, and the line represents model predictions with 95% confidence intervals

Higher levels of insect body δ^15^N did not translate to larger body sizes for weevils (multiple *R* ^2^ = 0.045, *F*‐statistic = 1.2, *df* = 26, *p* = .281; Figure 6a), nor for ground beetles (multiple *R* ^2^ = 0.010, *F*‐statistic = 0.28, *df* = 28, *p* = .599; Figure 6b). Similarly, insect body size did not vary with distance from the river for any species of weevil or ground beetle (Table 2). Only species and sex affected insect body size, with male ground beetles slightly smaller than females. Estimates of putative salmon‐derived nitrogen showed that weevils and ground beetles collected at 0 m acquired approximately 10.7% and 7.5% of their nitrogen from salmon, respectively.

## 4 DISCUSSION

We found elevated δ^15^N in both the soil and the bodies of herbivorous insects near a salmon‐bearing river. Despite this enrichment, we did not find evidence of improved insect body condition; insect δ^15^N, percent body nitrogen, and distance from the river did not explain patterns of insect body size. While previous work has focused on isotopic values, our findings suggest that isotopes alone cannot be used to infer biological consequences for terrestrial consumers. Our findings also confirm and that there are important limitations to inferences that can be drawn from isotopes as a tracer (Feddern et al., 2019; Schindler & Smits, 2017; Walsh et al., 2020).

The increase in soil δ^15^N close to the river matches the expectation from the presence of accumulated salmon carcasses and predator activity close to the banks (Ben‐David et al., 1998; Gende et al., 2007; Harding et al., 2019). Previous research has found elevated soil and plant δ^15^N in areas heavily used by predators such as bears and wolves, as well as areas nearer to stream banks (Ben‐David et al., 1998). δ^15^N is also correlated with the presence of transplanted salmon carcasses, and it attenuates with distance from the stream bank (Feddern et al., 2019; Hocking & Reynolds, 2012). This soil δ^15^N gradient may be affected by other factors that affect nitrogen cycling and isotopic fragmentation, such as riparian productivity and plant–mycorrhizal associations (Hocking & Reynolds, 2011). Soil moisture may also affect ^15^N (Schindler & Smits, 2017) through processes such as denitrification in anaerobic water‐logged soils (Pinay et al., 2003). However, we found no correlation between soil moisture and soil δ^15^N. Thus, while soil moisture alone cannot explain our results, we cannot determine what proportion of the soil δ^15^N gradient was due to salmon.

Weevil body δ^15^N levels also increased with proximity to the river, though this was not true for ground beetles. Weevil larvae feed on coniferous root systems in the soil with limited mobility (Condrashoff, 1966), so they may be influenced more strongly by the quality of food at the place where they were oviposited. Adult weevils feed on plants, which can be nitrogen‐rich in the vicinity of salmon‐supporting rivers (Helfield & Naiman, 2001; Hocking & Reynolds, 2011; Wilkinson et al., 2005). In contrast, ground beetle larvae and adults are active predators which feed on invertebrates such as slugs, snails, and insect larvae (Bursztyka et al., 2018; Digweed, 1994; Mair & Port, 2001). Adults are highly mobile, with the ability to walk hundreds of meters per day or several hectares per season (Baars, 1979; Lagisz et al., 2010). Ground beetle diets, as reflected by δ^15^N, would therefore be integrated over a large area and would be less likely to match the distance from the stream where the beetles were caught than would be the case for weevils.

Counter to our expectations, insect body size was not correlated with body δ^15^N content nor with distance from the salmon‐bearing river. One possible explanation is that nitrogen‐15 does not increase the total amount of nitrogen available for growth and reproduction. As suspected, when we investigated this possibility, we found that body nitrogen‐15 was not correlated with total percent nitrogen for either trophic level. This suggests that nitrogen‐15 did not increase the overall nitrogen content of these organisms. Without an associated increase in total available nitrogen, the presence of high δ^15^N may not indicate a biological advantage for individual insects. However, salmon may contribute other nutrients, minerals, or lipids that we were unable to detect by examining only δ^15^N (Olsen, 1999).

Insect percent nitrogen content may not have increased with nitrogen‐15 enrichment if the terrestrial insects next to the stream (0 m) were not nitrogen limited compared with those farther away (50 m). While we observed a gradient in δ^15^N in soils and herbivorous weevils with distance from the river, the entire study area may be enriched in δ^15^N due to its location at a salmon site. For example, work by Vizza et al. (2017) showed that riparian invertebrates at salmon streams have elevated levels of δ^15^N when compared to non‐salmon streams, likely reflecting the historical influence of salmon subsidies. This study reported several orders of magnitude fewer adult spawners (5–14 individuals) over a spawning channel of similar length, yet reported similar percentages of total nitrogen uptake by streamside invertebrates (8%–10%) (Vizza et al., 2017). However, work by Hocking and Reimchen (2002) estimates much higher percentages of marine‐derived nutrients in weevils (~49%–72%) and carabids (~31%–47%) on nearby streams in our study area. This discrepancy may reflect a violation of our assumption that no carcasses reach 50 m, meaning that we are underestimating the uptake of marine‐derived nutrients in the beetles collected for this study. However, despite different ecological settings and magnitudes of salmon influence, studies in both high elevation inland and low elevation coastal watersheds demonstrate that stable isotope ratios do not reflect metrics of body condition.

Organisms may also be obtaining nitrogen directly or indirectly through mycorrhizal associations. Studies by Hocking and Reynolds (2011, 2012) showed that while percent leaf nitrogen usually increased with δ^15^N, this is not true for nutrient‐poor indicator plants (especially those that are ericaceous), where nitrogen acquisition is more likely dependent upon mycorrhizal fungi. This may influence the nitrogen intake of weevils, whose larvae feed on Douglas‐fir (máwál̓as) (*Pseudotsuga menziesii*) and Western Hemlock (lúq̓vás) (*Tsuga heterophylla*) in our study area (along with a variety of other riparian plants as adults) (Condrashoff, 1966), but also the predatory ground beetles, whose prey may feed on common plants with ericoid and non‐ericoid mycorrhizal associations, including false azalea (*Menziesia ferruginea*), blueberry (sík̓vás) (*Vaccinium* spp.), and bunchberry (ƛ̓ṃ́k̓vúlí) (*Cornus canadensis*). Regardless of these potential mechanisms, the lack of increased nitrogen content and larger body sizes in terrestrial insects enriched with δ^15^N shows no evidence of a salmon‐derived benefit for these organisms via nitrogen subsidies, unless there is a historical benefit to the study area overall. We are unable to detect the presence of a historical benefit or legacy, as this would involve comparisons to streams without spawning salmon. There may also be temporary subsidies to organisms during the spawning season, which we could not measure with our study design.

These findings suggest that despite elevated δ^15^N in the environment near a salmon stream and some assimilation of salmon‐derived nitrogen in terrestrial insects, body condition of these insects may not necessarily be improved. Body size affects the reproductive success of both male and female beetles (Fox et al., 2007; Stillwell & Fox, 2009), and in females, it is strongly correlated with fecundity (Honek, 1993; Juliano, 1985; Kajita & Evans, 2010). Additionally, insect body size is related to reduced mortality from desiccation and starvation, as well as increased intraspecific competition success (Chown & Gaston, 2010). Therefore, larger body size may indicate superior health and fitness, and is considered a proxy for improved body condition. The body condition of beetles, particularly their reproductive potential, has been shown to reflect both the habitat quality (Barone & Frank, 2003; Bommarco, 1998) and the food availability to juvenile stages (Dmitriew & Rowe, 2011; Reaney & Knell, 2015; Van Dijk, 1994). Here, we find no such link between nitrogen‐enriched habitat and improved body condition of ground‐dwelling beetles, suggesting nutrients from salmon may not translate to increased survival and reproduction of terrestrial insects at a local scale, though we cannot rule out potential effects on larval growth rates (e.g., Reaney & Knell, 2015).

## 5 CONCLUSION

While isotopes such as δ^15^N can indicate nutrient enrichment, including from salmon carcasses in riparian zones, we did not find linkages with percent nitrogen nor adult body size as an indicator of body condition in the weevils and carabid beetles we studied. This suggests that isotopic tracers cannot be reliably used to infer impacts of cross‐ecosystem subsidies on individual organisms. Instead, this valuable method of tracing nutrient flows needs to be coupled with direct measures of ecological consequence.

## CONFLICT OF INTEREST

The authors declare no conflict of interest.

## AUTHOR CONTRIBUTIONS

**Nicola F. Rammell:** Conceptualization (equal); Data curation (lead); Formal analysis (lead); Investigation (equal); Methodology (equal); Project administration (equal); Validation (lead); Visualization (equal); Writing‐original draft (lead); Writing‐review & editing (equal). **Allison M. Dennert:** Conceptualization (equal); Formal analysis (supporting); Investigation (equal); Methodology (equal); Project administration (equal); Supervision (supporting); Validation (supporting); Visualization (equal); Writing‐original draft (supporting); Writing‐review & editing (equal). **Christopher M. Ernst:** Conceptualization (supporting); Investigation (equal); Methodology (equal); Resources (equal); Validation (equal); Writing‐review & editing (equal). **John D. Reynolds:** Conceptualization (equal); Funding acquisition (lead); Investigation (equal); Methodology (equal); Project administration (equal); Resources (lead); Supervision (lead); Writing‐review & editing (equal).

## Data Availability

Data and reproducible code are freely available on GitHub at https://github.com/adennert/Beetle‐Paper.
